# Left lower lobe sleeve lobectomy for lung cancer using the Da Vinci surgical system

**DOI:** 10.1186/s13019-016-0453-8

**Published:** 2016-04-12

**Authors:** Yandong Zhao, Wenjie Jiao, Xiaoyang Ren, Liangdong Zhang, Tong Qiu, Bo Fu, Lei Wang

**Affiliations:** The Affiliated Hospital of Qingdao University, 16 Jiangsu Road, Qingdao, Shandong 266003 China

**Keywords:** Sleeve lobectomy, Lung cancer surgery, Robot-assisted lung resection, Minimally invasive surgery

## Abstract

**Background:**

Despite the robotic surgery is widely applied, sleeve lobectomy for lung cancer using the Da Vinci surgical system is still less performed. We described a sleeve lobectomy for adenocarcinoma located at the left lower lobe using the Da Vinci surgical system.

**Case presentation:**

A case of 57-year old female referred to our hospital. Computed tomography scan showed an occupation located at the left lower lobe and adenocarcinoma project from the lobe bronchus was diagnosed by bronchoscope examination. A sleeve lobectomy was performed using the Da Vinci surgical system and the postoperative recovery was uneventful.

**Conclusions:**

Robotic thoracic surgery is feasible to perform sleeve lobectomy inspite of inadequate experience.

## Background

Robot-assisted surgery using the da Vinci surgical system has became an extension of the minimally invasive lobectomy spectrum because of excellent operability under the clear vision of a three-dimensional high-definition camera. The robotic surgery for pulmonary resection is safe and efficient and has similar survival rates compared with the open and VATS approaches.

## Case presentation

**Fig. 1:**
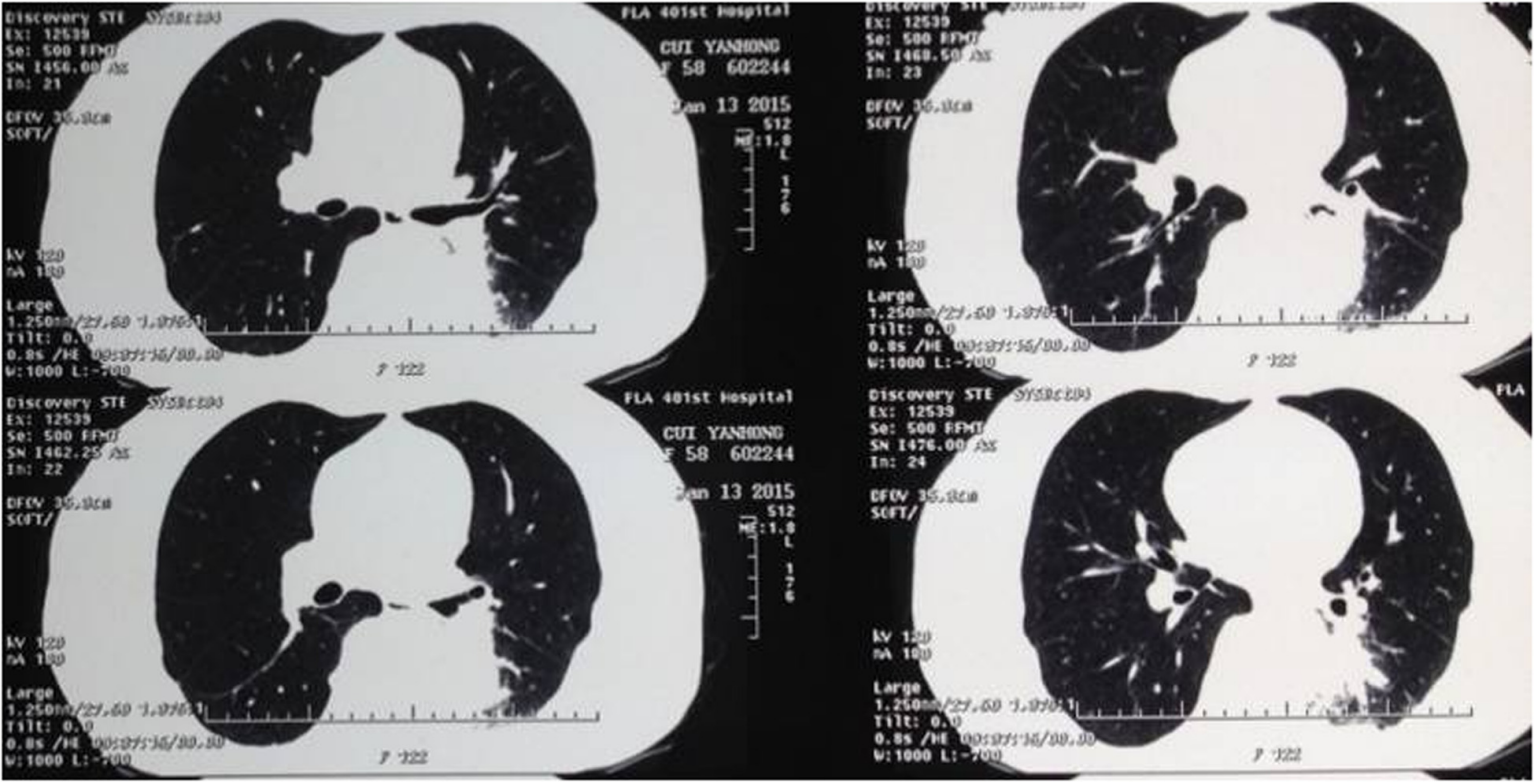
CT scan revealed a mass located at left lower lobe and the main and upper lobe bronchus were not involved

A 57-year old female, nonsmoker with a mass in left lung revealed by routine physical examination. CT scan revealed a 3 × 3 × 3 cm mass located at the dorsal segment of left lower lobe and projected into the lobe bronchus. Adenocarcinoma was diagnosed by bronchoscope examination (Fig. 1). The left main and upper lobe bronchus were not involved. The pulmonary artery and vein were free of tumor. Before the operation, the pulmonary function test, blood gas analysis, cardiac evaluation and basic examinations showed normal with no other comorbidities. The operation was performed using the Da Vinci surgical system by Dr. Jiao in Jan 29, 2015.

## Surgical technique

**Fig. 2:**
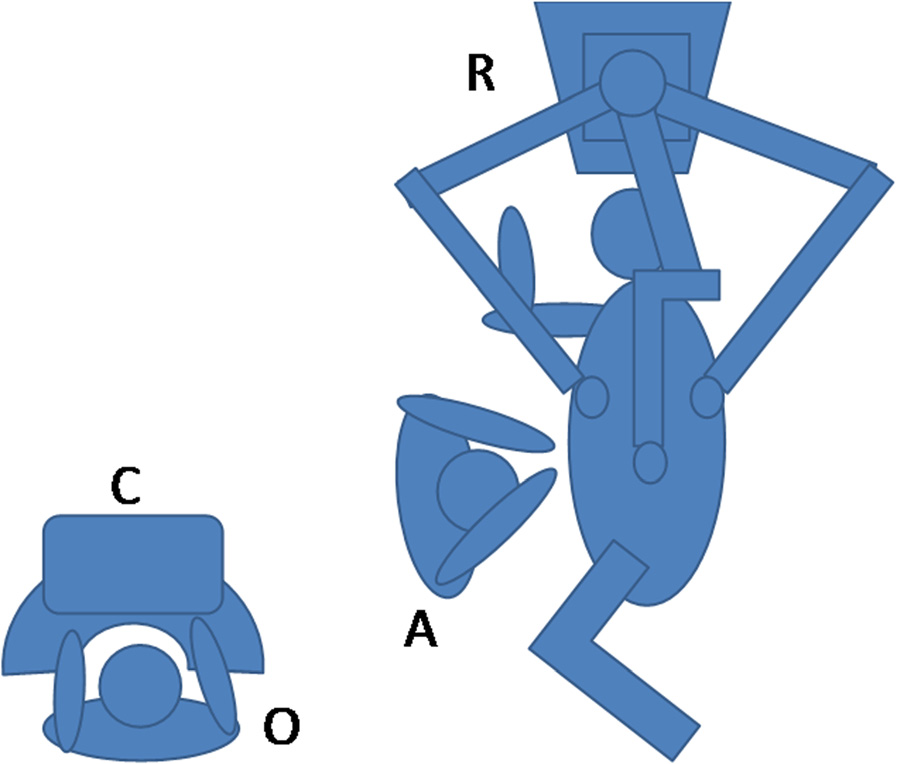
The position of robotic surgical system for left lower lobe sleeve lobectomy. (C: Console; R: Robotic system; O: Operator; A: Assistant)

The patient received general anesthesia with dual-lumen endotracheal intubation. Then positioned in the lateral decubitus position with the bed flexed to increase the intercostal space. A 12 mm port in the 7th intercostal space in the midaxillary line was placed as observing hole for camera. Other two incisions were made in the 4th intercostal space in the anterioraxillary line for the left arm and the 7th intercostals space in the subscapular line for the right arm respectively. The robotic surgical system was then brought into position and placed cephalad to the patient. One assistant on the patient’s right side (Fig. 2). A 25 mm incision in the 6th intercostal space in the anterioraxillary line for assistant instruments. We used an electrical hook in right arm to divide and a forceps in left arm for grasping.

**Fig. 3:**
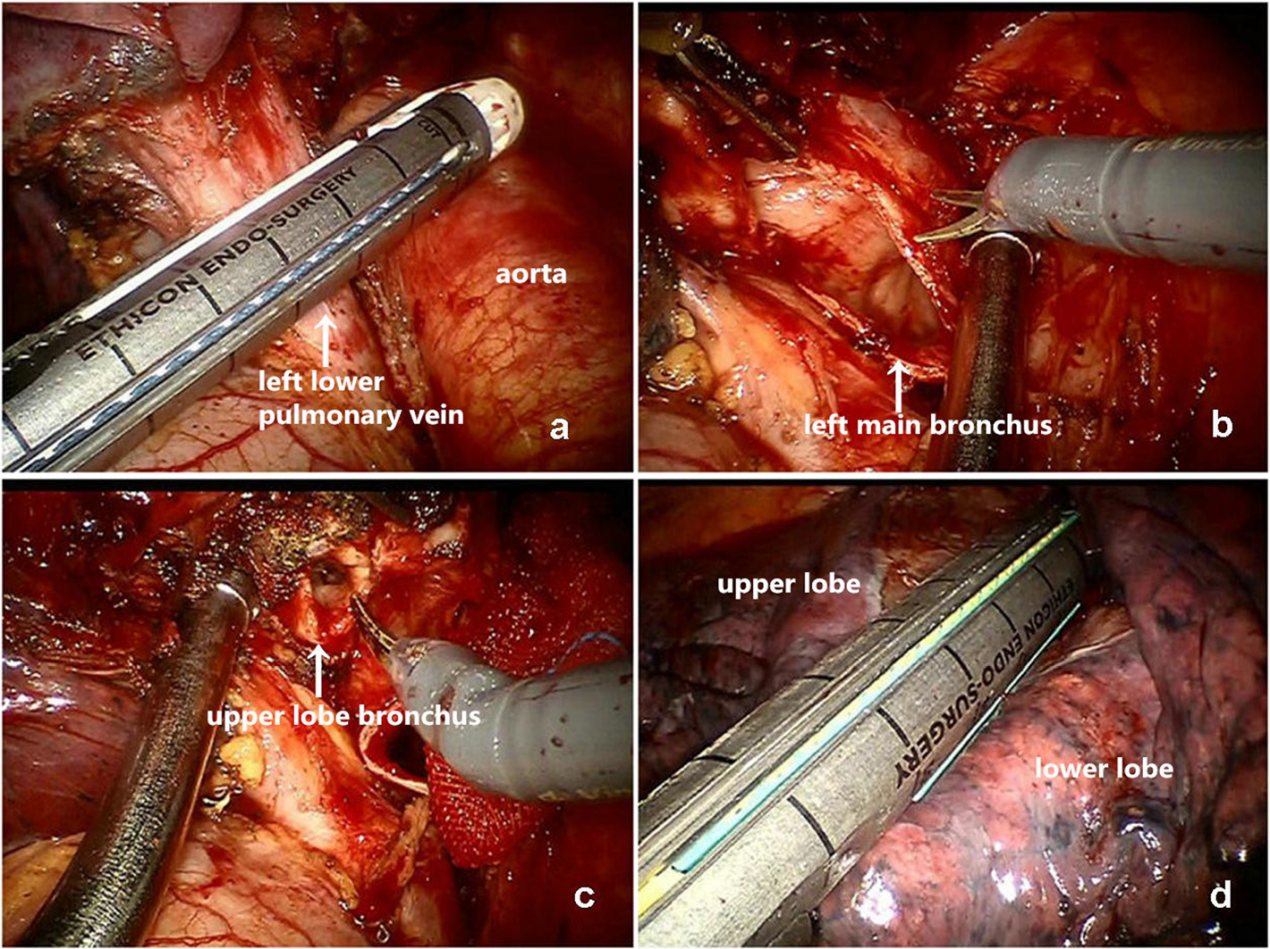
The resection of the left lower lobe. (**a** Transection of the left lower pulmonary vein with stapler; **b** The bronchus was divided; **c** transected the bronchus with scissors; **d** The fissure was divided by staplers.)

The mass was confirmed in the lower lobe. The surgical procedure started from releasing the inferior pulmonary ligament to bring enough mobility. The assistant retracted the lung up using orbicular-ovate grasping forceps and long curved suction to assist, followed by the dissection of the posterior and anterior wall of the hilum. Then mobilization and transection of the left lower pulmonary vein using a 2 mm stapler (Endo GIA™ 45 mm). The bronchi were encircled and then divided. The operator made a cut as marker at the pars cartilaginea with electrical hook and then transected the left main and upper lobe bronchus with scissors respectively. The fissure was divided by staplers. The branchs of the artery for lower lobe were dissected and stapled (Fig. 3).

**Fig. 4:**
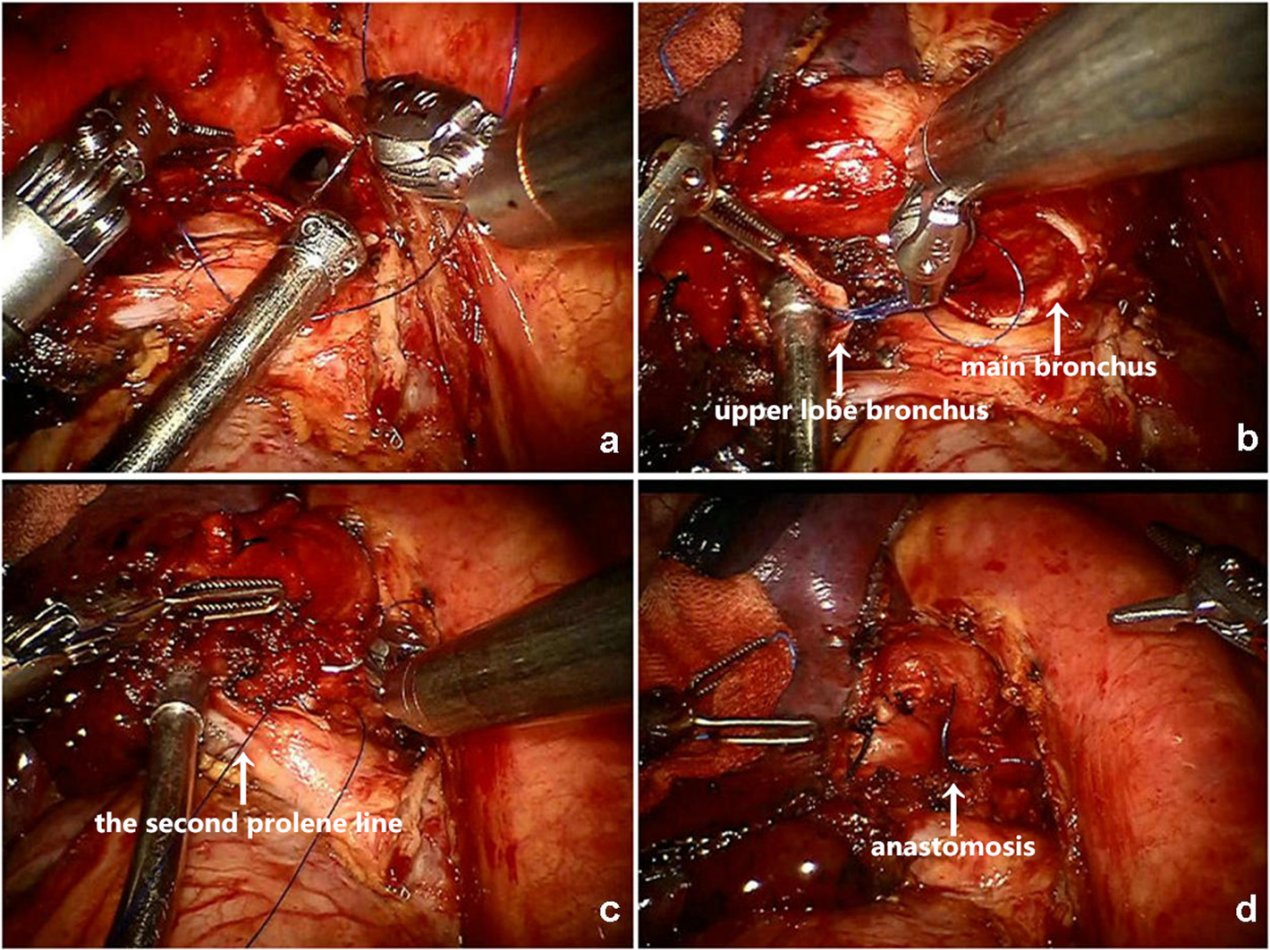
The anastomosis of bronchus. (**a** Running sutures from proximal of the anterior wall of the pars cartilaginea; **b** Needle running of the upper lobe bronchus; **c** Anastomotic sutures were tied to make the two ends get close; **d** The final knot was made at the posterior wall.)

The specimen was then removed in a glove. Negative margins were confirmed by frozen pathology examination. Systematic lymph node dissection was then performed. Then we changed instrument in right arm with needle holder. The end-to-end bronchial anastomosis was performed between the left main bronchus and upper lobe bronchus subsequently. A single continuous suture with 3/0 Prolene (Ethicon) was utilized. With about the length of 30 cm in thorax for anastomosis, the other end of prolene line was pulled out by the assistant in appropriate strength. We started from the anterior wall of the pars cartilaginea with continuous suture. Every stitch was followed by tightened up with forceps in left arm. The prolene line was broken up when the anterior wall almost finished. We brought in another line and continued with the residual sutures. The later one was cut into a length of 10 cm. A first knot was made between the two ends of the lines to ensure the continuity. Then the following part was finished with the later line continuously. The final knot was made at the pars cartilaginea between the two other ends of the lines (Fig. 4). We used 2 prolene lines in one anastomoses ultimately. Leak testing was conducted, in which no leakage was detected. The bronchial anastomosis was covered with an anterior mediastinum fat flap.

**Fig. 5:**
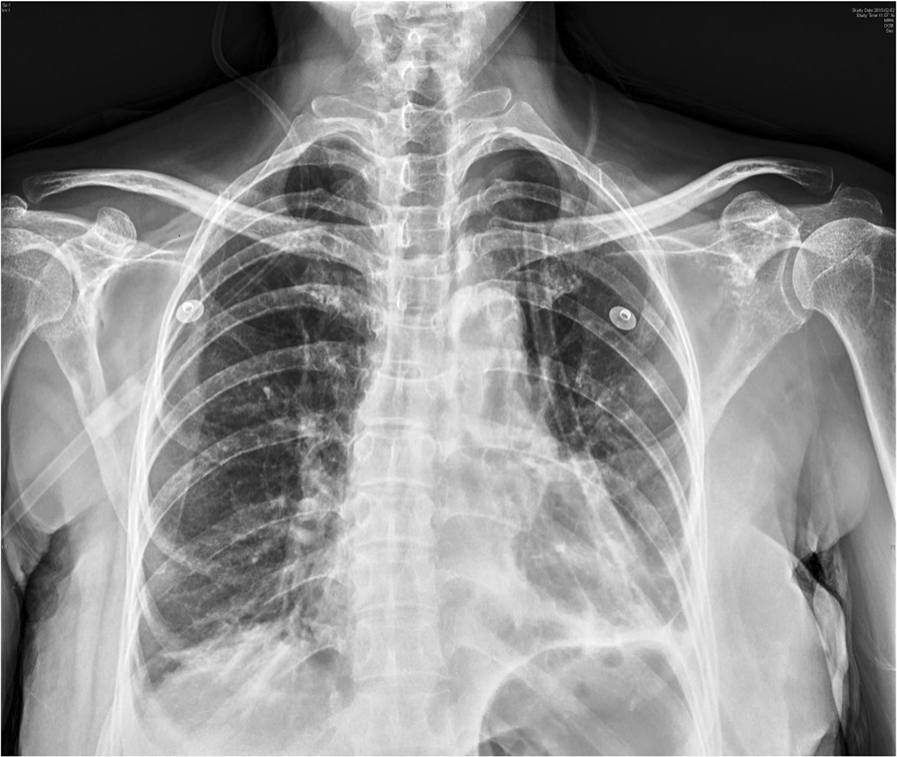
Chest X-ray of the 1st postoperative day

**Fig. 6:**
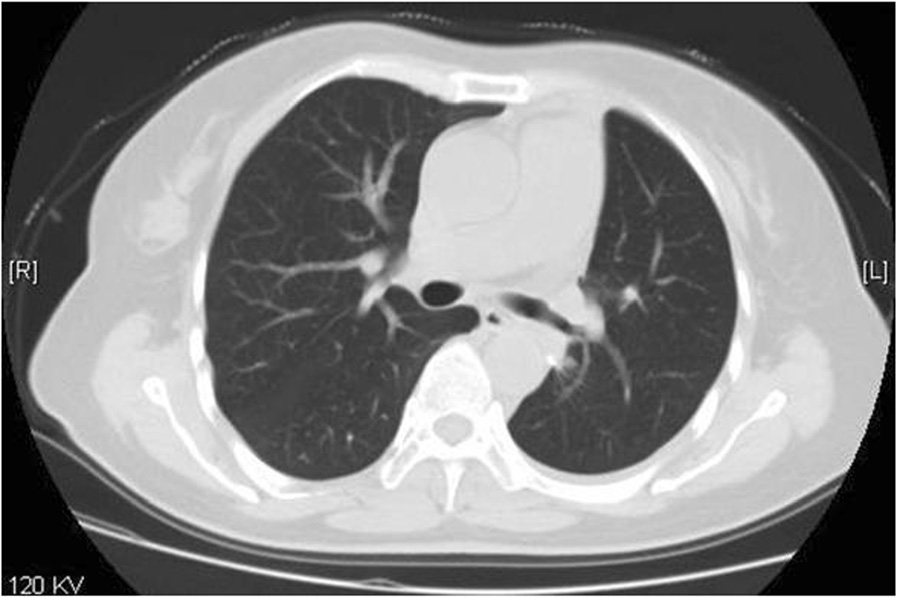
Chest CT scan at 1 month after operation

**Fig. 7:**
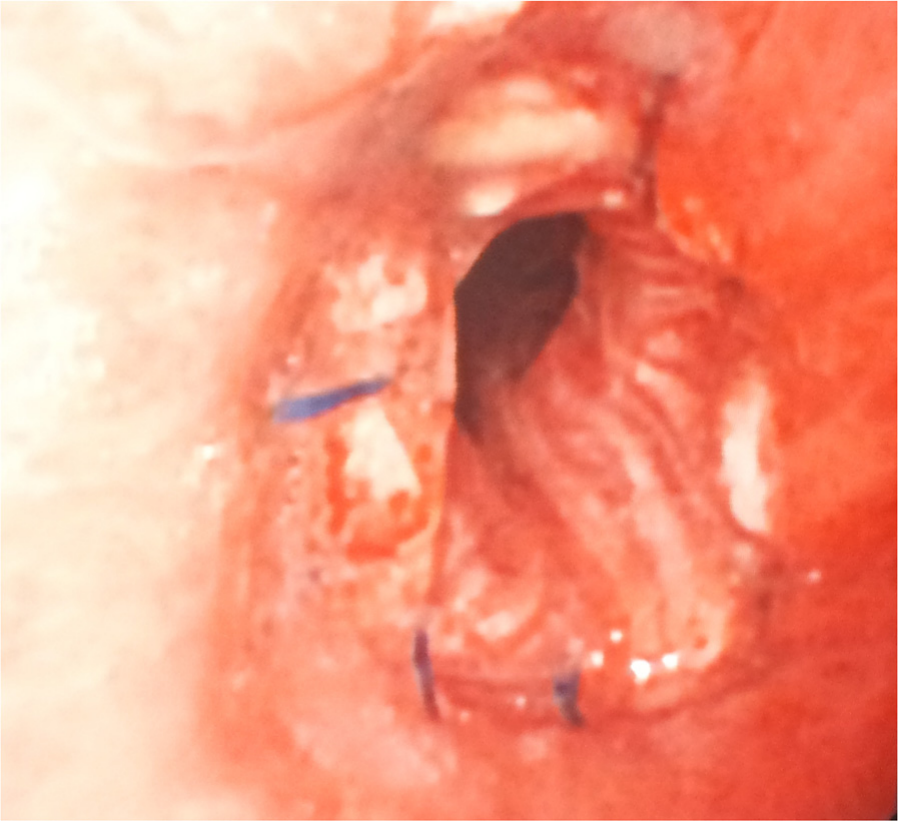
The anastomosis in bronchoscopy at 2 months after operation

The estimated blood loss was 100 ml. Postoperative chest X-rays showed no signs of atelectasis (Fig. 5). And the chest tube was removed on the 3rd postoperative day. The patient was discharged on the 7th postoperative day. The postoperative recovery was uneventful and the CT scan at 1 month showed no abnormal signs (Fig. 6). Bronchoscopy was made at 2 months to reveal no signs of anastomotic stricture (Fig. 7). The patient has been followed for 13 months without any signs of recurrence or metastasis.

## Comment

The first use of robotic system for primary lung cancer was reported by Melfi [1] in 2002. Due to safety concerns, the surgery was converted to thoracotomy in two of five patients. As the surgeon can acquire a high-resolution, three-dimensional and magnified view of the operative field with wristed instruments providing articulated movements, the performance of urologic, gynecologic and cardiac operations has been proven to be safe and feasible. Robotic lobectomy is being offered increasingly to patients [2]. The third (Si) generation da Vinci system has developed series of new robotic instruments, allow control of various operative steps from the console, especially for needle running which is essentially utilized in plastic surgery.

Sleeve resection was used to be concerned as a contraindication for minimally invasive surgery and few studies were reported. The conception was widely accepted after the first report of a VATS sleeve lobectomy given by Santambrogio in 2002 [3]. Robotic sleeve resection is an extension of the minimally invasive sleeve lobectomy spectrum. Ishikawa and colleagues described the first report on a robotic sleeve lobectomy in a human cadaver in 2006 [4]. However, few reports published on robotic-assisted sleeve lobectomy. A combined robotic and VATS approach for a minimally invasive sleeve lobectomy in a right upper lobe was reported by Schmid [5] in 2011.

The considerations of minimally invasive sleeve lobectomy included to reach the area by a two-dimensional image and the patent suturing of the anastomosis. And we believe that the developments on robotic system are important steps answering the considerations of the surgical technique. Another difficulty is the distance between the two ends of bronchus may require high tension on the sutures. We chose 2 prolene lines with continuous running sutures, however interrupted sutures was much more popular and a single continuous running involved a risk of breakage. In our experience, the anastomosis could be tightened up finally for smooth prolene, which gave an enough space for former sutures running. The length of the line in thorax was calculated as about 30 cm to avoid line twist.

Robotic thoracic surgery offers specific advantages over VATS with accuracy and flexibility especially in bronchial plastic surgery. In the future, to make a deep investigation of the benefit, long-term survival studies in patients with lung cancer should be carried out subsequently.

## Conclusion

Robotic thoracic surgery is feasible to perform sleeve lobectomy inspite of inadequate experience.
